# Early-Life Exposure to the Mycotoxin Fumonisin B1 and Developmental Programming of the Ovary of the Offspring: The Possible Role of Autophagy in Fertility Recovery

**DOI:** 10.3390/toxics11120980

**Published:** 2023-12-03

**Authors:** Awadh Alhelaisi, Abdulkarem Alrezaki, Saber Nahdi, Waleed Aldahmash, Saleh Alwasel, Abdel Halim Harrath

**Affiliations:** Department of Zoology, College of Science, King Saud University, Riyadh 11451, Saudi Arabia; alhelaisi@gmail.com (A.A.); aayr1978@gmail.com (A.A.); nehdisabeur@gmail.com (S.N.); waldahmash@ksu.edu.sa (W.A.); salwasel@ksu.edu.sa (S.A.)

**Keywords:** mycotoxins, fumonisin B1, ovary, female fertility, steroidogenesis, autophagy

## Abstract

Mycotoxins are produced by more than one hundred fungi and produce secondary metabolites that contaminate various agricultural commodities, especially rice and corn. Their presence in the food chain is considered a serious problem worldwide. In recent years, a link between exposure to mycotoxins and impaired fertility has been suggested. Consequently, it has become vital to investigate the interactive effects of these mycotoxins on ovarian function. In this study, we investigated the intergenerational effects of the mycotoxin fumonisin B1 (FB1) on ovarian structure and function. Virgin Wistar albino female rats were separated into control and FB1 treatment groups and examined from day 6 of pregnancy until delivery (20 and 50 mg/kg b.w./day). The obtained female rats of the first (F1) and second generations (F2) were euthanized at 4 weeks of age, and ovary samples were collected. We found that the ovary weight index increased with the high dose of the treatment (50 mg/kg b.w./day) among both F1 and F2, in a manner similar to that observed in polycystic ovary syndrome. As expected, FB1 at a high dose (50 mg/kg b.w.) reduced the number of primordial follicles in F1 and F2, leading to an accelerated age-related decline in reproductive capacity. Moreover, it reduced the fertility rate among the F1 female rats by affecting follicle growth and development, as the number of secondary and tertiary follicles decreased. Histopathological changes were evidenced by the altered structures of most of the growing follicle oocytes, as revealed by a thinning irregular zona pellucida and pyknosis in granulosa cells. These findings are concomitant with steroidogenesis- and folliculogenesis-related gene expression, as evidenced by the decrease in CYP19 activity and estrogen receptor beta (ESR2) gene expression. Additionally, GDF-9 mRNA levels were significantly decreased, and IGF-1 mRNA levels were significantly increased. However, the results from the ovaries of the F2 treatment groups were different and unexpected. While there was no significant variation in CYP19 activity compared to the control, the ESR2 significantly increased, leading to stereological and histopathological changes similar to those of the control, except for some altered follicles. The hallmark histological feature was the appearance of vacuolar structures within the oocyte and between granulosa cell layers. Interestingly, the autophagic marker LC3 was significantly increased in the F2 offspring, whereas this protein was significantly decreased in the F1 offspring. Therefore, we suggest that the promotion of autophagy in the ovaries of the F2 offspring may be considered a recovery mechanism from the effect of prenatal FB1 exposure. Thus, autophagy corrected the effect of FB1 during the early life of the F1 female rats, leading to F2 offspring with ovarian structure and function similar to those of the control. However, the offspring, treated female rats may experience early ovarian aging because their ovarian pool was affected.

## 1. Introduction

Mycotoxins are chemicals produced by fungi that have a highly toxic impact on human and animal health, even at low concentrations [1]. They contaminate at least 25% of the global food supply [2], and that percentage is expected to increase with climate change [3,4]. The most common toxigenic fungal species are in the genera Aspergillus, Penicillium, and Fusarium [5]. The population structure of these microorganisms in foods depends on factors such as temperature, gas composition, and water availability [5,6]. Fumonisins are mycotoxins produced by *Fusarium verticillioides* and *Fusarium proliferatum* that contaminate some plants and their food products, mainly corn crops, and many other plants, such as sorghum, cowpea, asparagus, rice, and wheat [7]. Thus, regions where corn crops are used as dietary staples are highly exposed to fumonisin B1 (FB1), B2 (FB2), and B3 (FB3) [8,9].

It has been described that FB1 exposure is associated with esophageal cancer [10,11], porcine pulmonary edema [12,13], liver and kidney toxicity [14,15], and liver and kidney cancer [16,17] in different animal species. Additionally, pregnant animals fed FB1-contaminated feed have displayed growth delays, delayed or incomplete ossifications, cleft palate or hydrocephalus, and fetal death [18]. Neural tube defects (NTDs) due to fumonisin exposure, including spina bifida, exencephaly and craniorachischisis, or meningomyelocele, have been reported [6,18].

To date, very few studies have focused on the effect of FB1 on female reproduction. These studies suggested the role played by FB1 in affecting female fertility, as dietary concentrations of FB1 reduced serum gonadotropin levels and lowered fertility without inducing histopathological changes in the ovaries [19]. Furthermore, it has been reported that FB1 exposure leads to granulosa cell proliferation and affects progesterone production induced by FSH plus IGF-I [20]. However, there is still a need to explore the interactive effects of FB1 on ovarian function and female fertility in detail, as the exact underlying mechanism is unfamiliar. In this study, we provide comprehensive knowledge of the intergenerational effect of FB1 on the structure and function of the ovary. In particular, histopathological changes, follicular development, and folliculogenesis- and steroidogenesis-related genes were investigated. We also illustrate the role of autophagy as a recovery mechanism in the ovaries of the second generation to protect against the prenatal effect of FB1.

## 2. Results

### 2.1. Fumonisin B1 Changes the Ovary Weight Index and Fertility Rate

**Figure 1:**
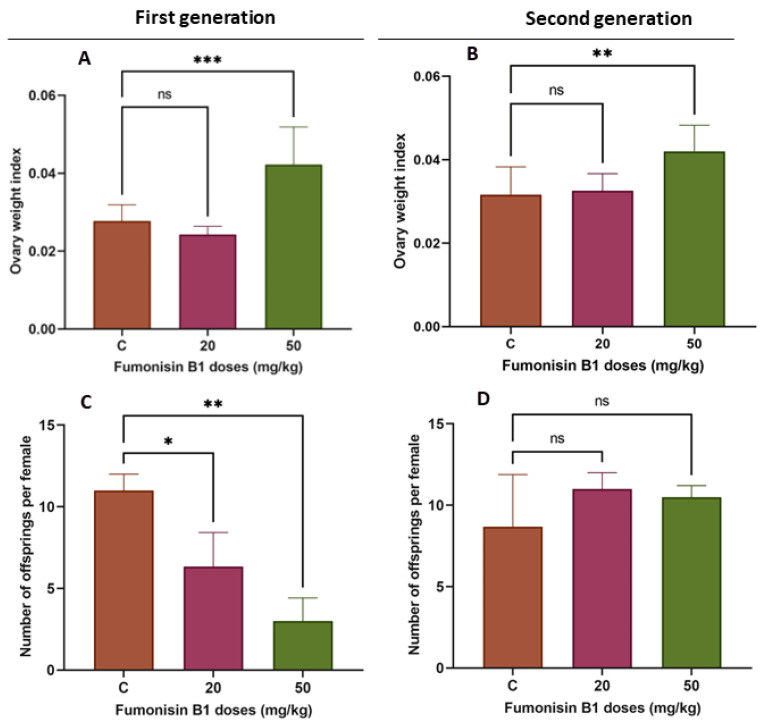
Ovary weight index in both the F1 (**A**) and F2 (**B**) treatment groups in comparison to the control group. There was a significant increase in the ovary weight index in the rats from the high-dose (50 mg/kg) FB1 treatment group but a non-significant effect in the low-dose (20 mg/kg) group. The number of offspring per female in F1 (**C**) significantly decreased compared to that in the control group; yet, among F2 female rats (**D**), there was no significant difference in the fertility rate compared to the control group. (*) indicates a *p* value < 0.05, (**) indicates a *p* value < 0.01, (***) indicates a *p* value ≤ 0.001.

In the present study, an increased ovarian weight index was found in the 50 mg/kg treated group in both the first and second generations (Figure 1A, *p* < 0.05), indicating that fumonisin B1 may affect ovarian development. However, the lower dose (20 mg/kg) did not show any significant effects (Figure 1A).

To assess the influence of fumonisin B1 on fertility, the number of offspring was evaluated. The results showed that fumonisin B1 exposure significantly decreased the number of offspring in the first generation in both treatment groups (20 and 50 mg/kg) but did not significantly affect the number of offspring in the second generation (Figure 1B).

### 2.2. Fumonisin B1 Alters the Number of Follicles

**Figure 2:**
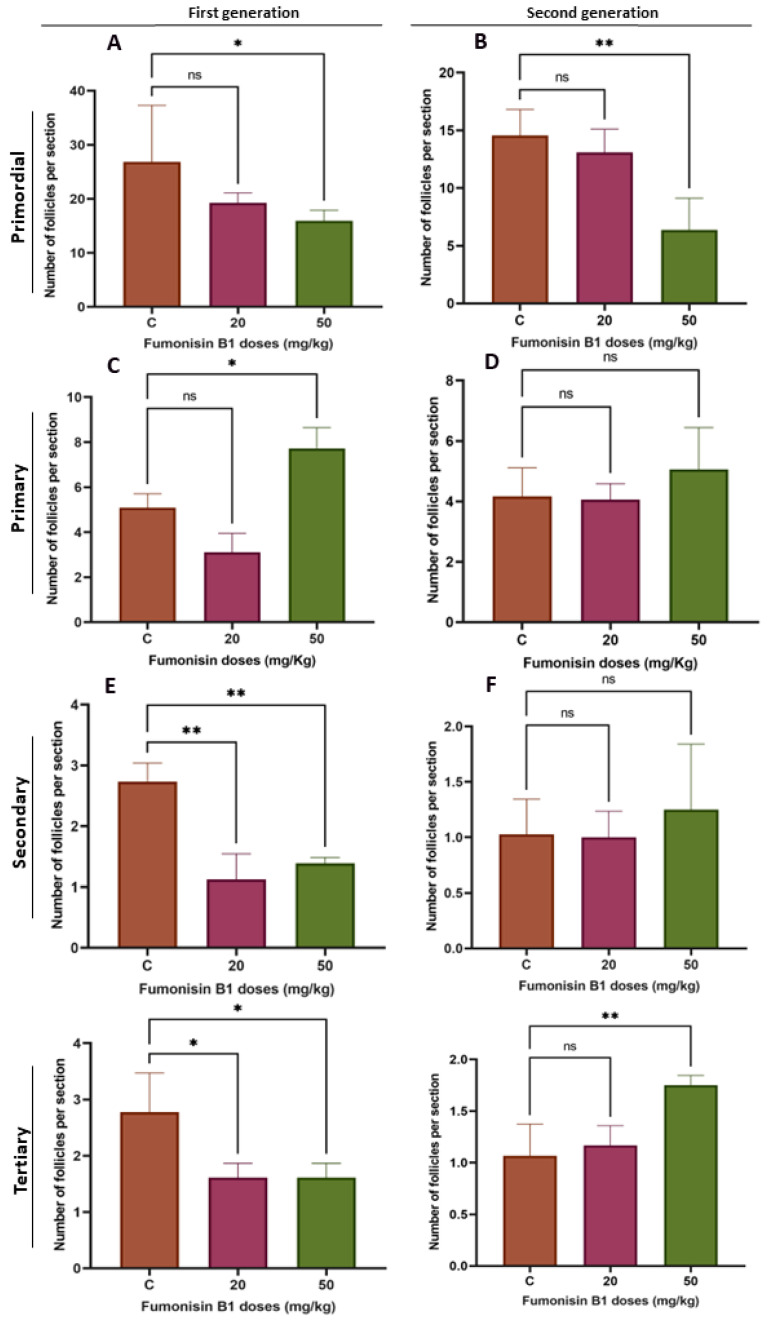
Primordial follicles in both F1D2 and F2D2 were significantly decreased (**A**,**B**). However, there was no significant decrease in F1D1 and F2D1. The number of primary follicles in F1D2 increased significantly. No changes were noted in F1D1, F2D1, or F2D2 (**C**,**D**). The number of secondary follicles in groups F1D1 and F1D2 significantly decreased, whereas no changes within groups F2D1 and F2D2 were observed (**E**,**F**). Similarly, the number of tertiary follicles in groups F1D1 and F1D2 significantly decreased compared to that in the control group, while a significant increase was observed in F2D2. D1: 20 mg/kg; D2: 50 mg/kg. (*) indicates a *p* value < 0.05, (**) indicates a *p* value < 0.01.

To explore the effect of fumonisin B1 on ovarian follicles, the number of primordial, primary, secondary, and tertiary follicles was evaluated. We found that the proportions of primordial follicles in the female rats treated with a high dose of FB1 (50 mg/kg) among both F1 and F2 were significantly decreased, whereas there was no decrease in the low-dose treatment groups (20 mg/kg) (Figure 2A). Additionally, the number of primary follicles in the 50 mg/kg F1 treatment group increased significantly, whereas there were no significant changes in the 50 mg/kg F2 treatment group or in the 20 mg/kg F1 and F2 treatment groups (Figure 2B). A decrease in the number of secondary follicles was also found in the F1 female rats (20 and 50 mg/kg treated group) but not in the F2 treatment group of female rats (Figure 2C). Last, the number of tertiary follicles in the F1 treatment group was significantly decreased compared to that in the control group and significantly increased in the 50 mg/kg F2 treatment group (Figure 2D).

### 2.3. Fumonisin B1 Alters Oocyte Structure and Follicle Growth Histology

**Figure 3:**
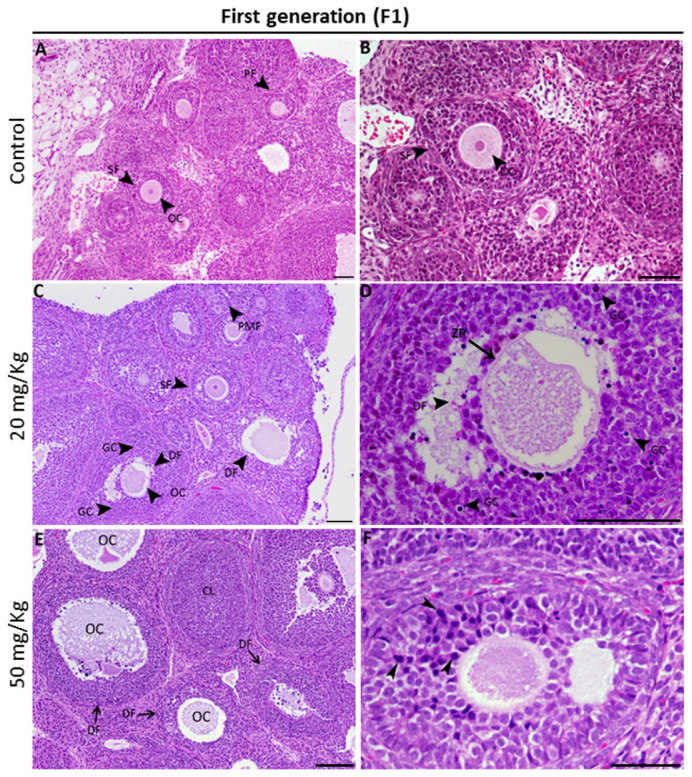
Representative micrographs of H and E-stained ovary tissue sections from the control female rats (**A**,**B**) and the exposed female rats of the first generation (**C**–**F**). (**A**) In the control offspring, the ovaries had a definite and typical histological organization with different types of growing follicles. (**C**–**F**) The hallmark morphological feature of the ovaries from the group prenatally exposed to FB1 was an increased number of degenerative follicles (DF) compared to the control. These degenerative altered follicles generally have an altered oocyte with an irregular zona pellucida (**D**) and an increased number of pyknotic granulosa cell nuclei (arrowheads in **D**). CL: corpus luteum; DF: degenerative follicle; GC: granulosa cell; OC: oocyte; PM: primary follicle; PMF: primordial follicle; SF: secondary follicle. Scale bar = 60 µm.

**Figure 4:**
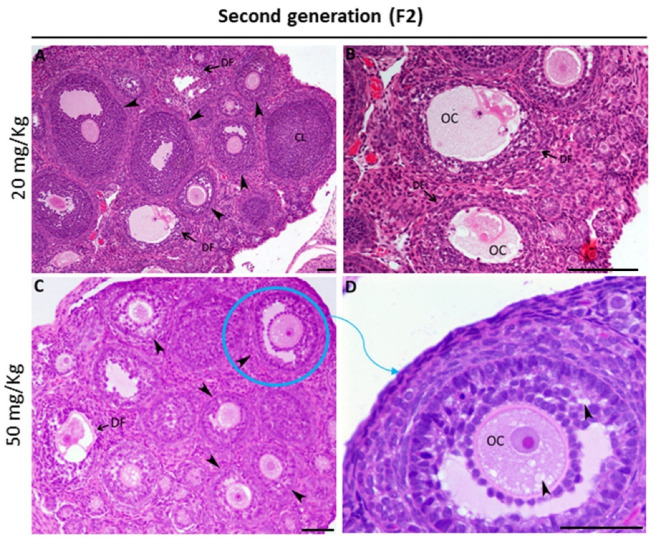
Representative micrographs of H and E-stained ovary tissue sections from the exposed female rats of the second generation (**A**–**D**). (**A**,**B**) In the group prenatally exposed to 20 mg/kg FB1, most of the primordial and growing follicles were normal, although some disrupted follicles were found (DF). (**C**–**D**) However, the hallmark morphological feature of the ovaries from the group prenatally exposed to 50 mg/kg FB1 was the appearance of many vacuoles present within the oocytes and between the layers of granulosa cells (arrowheads in **D**). CL: corpus luteum; DF: degenerative follicle; OC: oocyte. Scale bar = 60 µm.

The influence of fumonisin B1 on the histology of the ovary was assessed by observing each follicle in the different groups (control and treated). We found that, compared to the control, in which a normal structure of the ovary was observed (Figure 3A,B), histological changes were observed at low and high doses of treatment. In fact, ovaries from F1 female rats treated with low and high doses showed alterations in the structures of most of the oocytes of the growing follicles (Figure 3C–F), as revealed by a thinning irregular zona pellucida (Figure 3D). The number of granulosa cells with a high number of pyknotic nuclei was higher than that in the control group (Figure 3E,F). However, the structure of the ovary from F2 female rats was unexpectedly similar to that of the control. In fact, with respect to the small number of altered follicles (Figure 4A,B), we found that most of the growing follicles were normal. Additionally, the ovaries from the high-dose treatment group of F2 female rats showed the presence of many vacuoles within the oocytes and between the layers of granulosa cells (Figure 4C,D).

### 2.4. Fumonisin B1 Affects Folliculogenesis- and Steroidogenesis-Related Gene Expression

**Figure 5:**
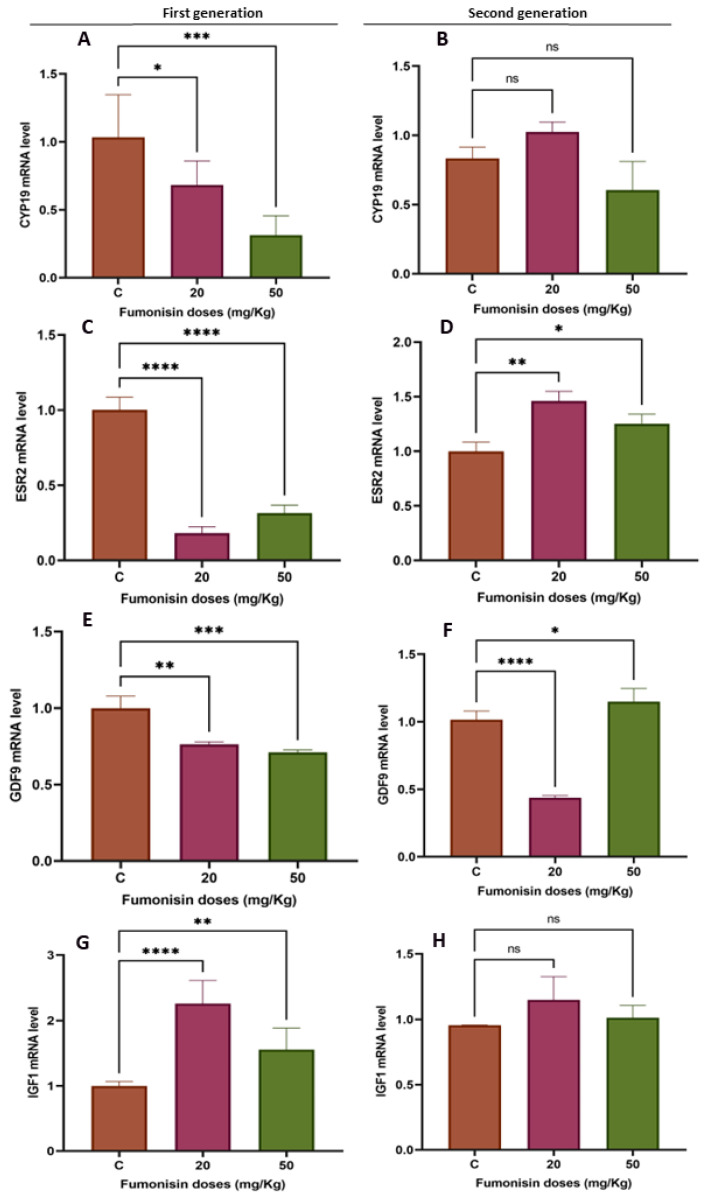
mRNA expression levels of some folliculogenesis- and steroidogenesis-related genes in the ovaries of rats in the treatment groups compared to the control group from the different generations. (**A**) CYP19 mRNA levels in the first generation; (**B**) CYP19 mRNA levels in the second generation; (**C**) ESR2 mRNA levels in the first generation; (**D**) ESR2 mRNA levels in the second generation; (**E**) GDF9 mRNA levels in the first generation; (**F**) GDF9 mRNA levels in the second generation; (**G**) IGF1 mRNA levels in the first generation; (**H**) IGF1 mRNA levels in the second generation. The values are presented as the mean ± S.E.M. (*) indicates a *p* value < 0.05, (**) indicates a *p* value < 0.01, (***) indicates a *p* value ≤ 0.001, and (****) indicates a *p* value ≤ 0.0001, ns: non-significant.

To evaluate the effects of fumonisin B1 on the expression of folliculogenesis- and steroidogenesis-related genes, we analyzed the expression of the CYP19, ESR2, GDF9, and IGF1 genes (Figure 5). We found that the mRNA expression of CYP19 and IGF1 was decreased in the treatment groups of the first generation, while no significant effect was observed in the treatment groups of the second generation. Additionally, while ESR2 mRNA expression significantly decreased in both of the treatment groups of the first generation, it significantly increased in the treatment groups of the second generation. The GDF9 mRNA levels significantly decreased in the 20 and 50 mg/kg treatment groups of the first generation, whereas their levels significantly decreased in the 20 mg/kg treatment group of the second generation and then significantly increased in the 50 mg/kg treatment group of the same generation. The IGF1 mRNA levels significantly decreased in the ovaries of the first generation, while no effect was found in the ovaries of the second generation.

### 2.5. The Role of Autophagy in Fumonisin B1 Treatment

**Figure 6:**
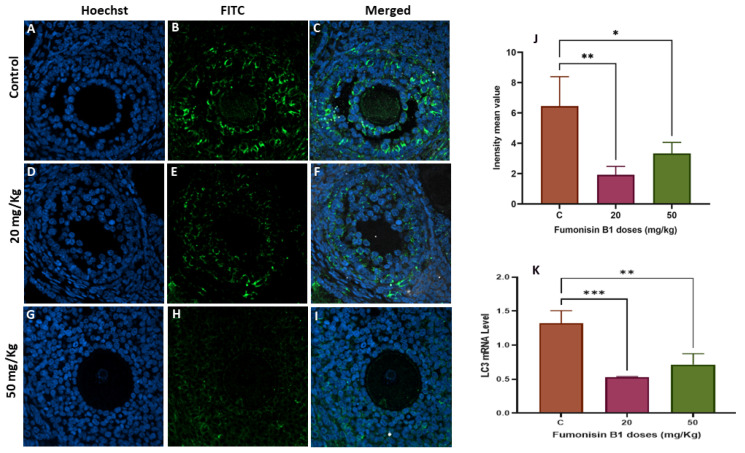
The autophagic marker LC3 in ovarian tissue from the first generation was evaluated using immunofluorescence staining in the control (**A**,**C**), treated with the secondary antibody FITC (**B**,**E**,**H**), 20 mg/kg FB1-treated F1 (**D**,**F**), and 50 mg/kg FB1-treated F1 groups (**G**,**I**). The relative fluorescence intensity (**J**) of LC-3 in the control and exposure groups was assessed with the Zen 3.1 service (ZEN lite) and quantified using the GraphPad Prism 9 program (GraphPad Software 10.1.1). The LC3 mRNA levels in the F1 FB1-treated groups compared to those in the control groups were determined using RT–PCR (**K**). Scale bar = 200 µm. (*) indicates a *p* value < 0.05, (**) indicates a *p* value < 0.01, (***) indicates a *p* value ≤ 0.001.

**Figure 7:**
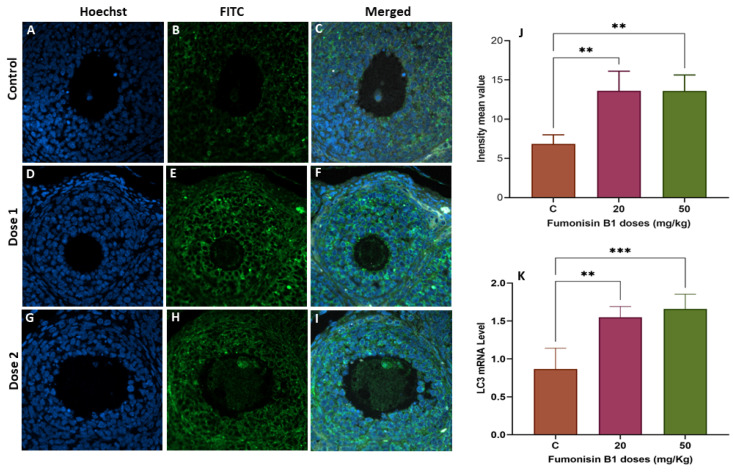
The LC3- autophagic marker in ovarian tissue from the second generation was evaluated using immunofluorescence staining in the control (**A**,**C**), treated with the secondary antibody FITC (**B**,**E**,**H**), 20 mg/kg FB1-treated group of F2 female rats (**D**,**F**), and 50 mg/kg FB1-treated group of F2 female rats (**G**,**I**). The relative fluorescence intensity (**J**) of LC-3 in the control and exposure groups was assessed with the Zen 3.1 service (ZEN lite) and quantified using the GraphPad Prism 9 program (GraphPad Software 10.1.1). The LC3 mRNA levels in the FB1-treated groups of F2 female rats compared to those in the control group were determined using RT–PCR (**K**). Scale bar = 200 µm. (**) indicates a *p* value < 0.01, (***) indicates a *p* value ≤ 0.001.3. Discussion.

To explore whether fumonisin induced autophagy, LC3 gene and protein expression was measured using RT–PCR and immunofluorescence staining, respectively. The results showed that the levels of LC3 protein were significantly decreased in the 20 and 50 mg/kg treatment groups of the first generation (Figure 6A–J), while a significant increase in LC3 protein was found in both of the treatment groups of the second generation (Figure 7A–J). To validate this finding, the expression levels of the autophagy-related gene LC3 were also detected. The results of the IF are aligned with those of the RT–PCR and showed that there was a significant decrease in LC3 mRNA levels in the 20 and 50 mg/kg groups of the first generation (Figure 6K), while a significant increase in LC3 mRNA levels was found in both groups of treatment in the second generation (Figure 7K).

Mycotoxins are produced by more than one hundred fungi that belong to the genera *Aspergillus*, *Penicillium*, and *Fusarium*. They produce secondary metabolites with low molecular weights that contaminate various agricultural commodities [21,22], especially rice and corn, which are widely used ingredients in food in developing countries [23,24]. Their occurrence in the food chain is considered a serious problem worldwide [25,26]. In particular, fumonisin B1, which was first isolated from *Fusarium* [27], mainly *F. verticillioides*, has been reported to be carcinogenic to humans [28,29] and may have a direct or indirect effect on the increased prevalence of some common diseases, such as female infertility, as a notable number of women are undergoing fertility treatment [30]. Consequently, it is vital to investigate the interactive effects of these mycotoxins on ovarian function.

## 3. Discussion

The characteristics and quality of the ovary greatly affect its function in reproduction; therefore, any disruption to ovarian function can be associated with an ovarian disorder, such as polycystic ovary syndrome [31]. Our results showed that the number of primordial follicles was significantly decreased in the F1 and F2 female rats treated with 50 mg/kg of fumonisin B1. It has been shown that the formation of primordial follicles can be influenced by several genes that control oocyte survival or apoptosis, thereby affecting the number of follicles formed [32,33]. Thus, FB1 affects the ovarian pool of healthy primordial follicles during early fetal life and is associated with a faster decline in ovarian function with aging. This finding is in agreement with previous studies reporting that prenatal exposure to environmentally relevant substances accelerates the age-related decline in reproductive capacity in the F1 generation [34,35]. Furthermore, the significant decrease in the number of primordial follicles in the 50 mg/kg treatment group of F2 female rats was associated with an increased number of tertiary follicles, suggesting that FB1 might cause early menarche by inducing early folliculogenesis. In fact, as the offspring of the second generation have a lower ovarian reserve of primordial follicles and, therefore, a shorter reproductive lifespan than those of the control, folliculogenesis was promoted early to maintain a high fertility similar to the control, leading to a successful reproductive lifespan. This hypothesis seems consistent with previous studies [36,37,38]. Thus, mothers of the first generation pass on a new phenotype that is better suited for the effect of FB1.

The high dose of FB1 significantly affected ovary weight among the two generations compared to the control. In fact, there was a significant increase in ovary weight at the higher doses for both the first and second generations compared to the control group. This finding is in agreement with previous reports showing that treatment with some substances, such as acrylamide, sodium fluoride, BPA, and nonylphenol, led to an increase in ovary weight [39,40,41,42]. Although cyclic fluctuations are connected to ovarian function during the estrous cycle, it is known that ovarian weight does not significantly change in normal rats. Consequently, any change in ovarian weight, whether positive or negative, should be viewed as a sign of ovarian dysfunction that may be caused by a variety of conditions, including persistent polycystic ovaries, oocyte and follicle depletion, luteal cyst development, and reproductive aging. In particular, the dominant luteinized follicle shrinks and ruptures in a normal cycle, but, in some circumstances, its cystic nature can persist during the luteal phase. This condition is known as “luteinized unruptured follicle syndrome (LUFS)”, which is an abnormal condition in which the follicle does not rupture during the luteal phase, causing cysts and leading to an increase in ovarian weight and a decrease in fertility [43,44]. Luteal cyst development has been described in spontaneously cycling women with unexplained infertility [45]. Thus, we suggest that high doses of FB1 may lead to the development of LUFS and, probably, to polycystic ovary syndrome (PCOS), the most common endocrine disorder in premenopausal women [46]. This finding is concomitant with the results of the steroidogenesis- and folliculogenesis-related gene expression in both treatment groups of the first generation. In fact, PCOS is thought to be caused by many intraovarian disturbances in steroidogenesis, including deficiency in the activity of CYP19, the enzyme that catalyzes the rate-limiting step in the biosynthesis of estrogens from androgens [47]. Thus, a decrease in the activity of this enzyme could be expected to result in a decrease in the expression of estrogen receptor beta (ESR2) and, therefore, increased ovarian androgen production and the development of PCOS [48,49]. In light of these facts, follicle growth and development of the first generation have been affected, leading to a decrease in the number of growing follicles, notably secondary and tertiary follicles. This may be explained by the degenerative follicles due to the vacuolized oocytes and disorganized granulosa cells with pyknotic nuclei, similar to the results reported in many previous studies [35,50,51]. As a result, the fertility rate of the first generation significantly decreased in both the 20 and 50 mg/kg treatment groups.

However, the results in the ovaries from the treatment groups of the second generation were different and unexpected. Indeed, while ovary weight significantly increased with the high dose of treatment, the steroidogenesis- and folliculogenesis-related genes did not decrease as they had among the female rats of the first generation. While there was no significant variation in CYP19, ESR2 was significantly increased. This result led us to think about the eventual role played by the autophagy process, as LC3 significantly increased in both groups of the second generation but significantly decreased in both groups of the first generation. The promoted autophagy among the second-generation ovaries may be considered a recovery mechanism from the effect of FB1. This finding is consistent with the stereological and histological results, as no significant difference was found in the fertility rate of the second generation compared to that of the control. Interestingly, the number of primary and secondary follicles of the treatment group was similar to that of the control group, and many vacuoles were observed within the oocytes and between the layers of granulosa cells. These vacuoles could be interpreted as autophagosome structures related to autophagy. In fact, proper functional autophagy is needed for the normal growth and development of follicles [52], and its occurrence is an Indicator of an adaptation to stress and can lead to reduced apoptotic cytotoxicity [53,54]. Its key role in maintaining normal cell homeostasis and preventing chronic cellular damage by removing toxins, damaged organelles, and unfolded proteins has been widely described [55,56]. Many previous studies have reported the association between autophagy and different ovarian disorders, including follicular cyst formation, metabolic abnormalities, and PCOS [57]. In particular, defective autophagy in ovarian cells leads to poor-quality oocytes, resulting in female infertility [58].

## 4. Materials and Methods

### 4.1. Ethical Statement

This study was approved by the Scientific Research Ethics Committee at King Saud University, Riyadh, Saudi Arabia (Reference No: KSU-SE-22-41) and carried out in accordance with the approved guidelines. All the experimental procedures are reported in compliance with the ARRIVE guidelines.

### 4.2. Study Design and Sampling

Thirty healthy pubertal virgin female Wistar-Albino rats (weighing 200–250 g) were housed separately in cages and kept in a facility with a standard laboratory chow diet and a 12–12 photoperiod at a temperature of 21 °C. The rats were then kept with male rats, and, when a white vaginal plug appeared on the cage flooring, mating was considered successful; that day was regarded as day 0 of gestation (GD 0). Thereafter, pregnant female rats were separated into three groups, and the following treatment regimen was followed from GD 6 to GD 21: 1) The first group of female rats (n = 10) received a gavage of distilled water and was designated as the control group; FB1 was administered orally to the second group of female rats (n = 10) at a dose of 20 mg/kg b.w./day; and FB1 was administered orally to the third group of female rats (n = 10) at a dose of 50 mg/kg b.w./day. The doses of treatment were selected based on previous studies [59,60]. Following parturition, we obtained the first generation of offspring from mothers who had been treated with FB1, which are referred to as the animals of the first generation (F1); the control group is referred to as the control group of the first generation (CF1). When the female F1 and CF1 offspring were 4 weeks old (before puberty), a fraction of them was moved into a clear plastic box with a carbon dioxide tube attached and a flow rate of 10 L/h for 10 min in preparation for euthanasia. The ovaries were cleaned, rapidly measured, and assigned to groups based on where they came from. The remaining female rats from F1 and CF1 were allowed to mature sexually and were mated with male rats. The second generation of offspring was obtained from the FB1-treated F1 mothers. These female rats are referred to as the FB1 offspring of the second generation (F2), while those obtained from the control group are referred to as the control group of the second generation (CF2). When the female F2 and CF2 offspring were 4 weeks old, they were euthanized, and their ovaries were sampled. The reproductive capacities of the groups were compared by calculating the ovary weight index (weight of the ovary/weight of the corresponding female rat) and fertility rate (number of offsprings/number of female rats (mothers)).

### 4.3. Histological Preparation

Neutral-buffered formalin (NBF 10%) was used to fix the ovary samples for a period of 24 h. The next day, they were embedded in paraffin and cut into sections with a thickness of 5–7 μm, collected on a hotplate, and transferred to glass slides containing warm (30 °C) water and albumin glycerol fixative for adhesion. Wrinkles were removed, and the sections were stained with hematoxylin and eosin for a histological study [36].

### 4.4. Immunofluorescence Staining and Confocal Microscopy

Immunostaining was performed as described in previous studies [35]. We placed slides containing tissue sections on a hot plate (60 °C) and deparaffinized them with xylene. They were then rehydrated and washed twice with distilled water and three times with 1× PBS. The slides were removed from the wash solution and dried. After drying, the sections were placed in a suitable container, permeabilized with 0.1% of Triton X-100 containing 0.1% of sodium citrate, and treated with a blocking buffer (1% BSA in PBS) at room temperature. The slides were placed in a humid box and incubated with a primary antibody solution (anti-LC3) (dilution 1:500) from DGpeptidesCo. Ltd. overnight at 4 °C on a flat surface, in the dark. The next day, the slides were washed four times with 1× PBS and treated with the secondary antibody FITC (dilution 1:2000) (ab6717, Abcam, Cambridge, UK) for 45 min at room temperature (RT), in the dark. Then, the slides were washed with PBS and a TE buffer before adding a Hoechst solution (diluted 1:15,000, Hoechst 33342, Life Technologies, Grand Island, NJ, USA). Finally, the sections were placed in a 50% glycerol/TE solution, and the edges were sealed with nail polish. The sections were observed and imaged for signal quantification with a spinning disk confocal microscope from Zeiss. The signal intensity for protein expression was analyzed with the Zen 3.1 service (ZEN lite) and quantified using the GraphPad Prism 9 program (GraphPad Software 10.1.1).

### 4.5. Analysis of Gene Expression

RNA was extracted using the RNeasy Mini Kit (Qiagen, Westburg, The Netherlands) with DNase treatment on columns using an RNase-free DNase kit (Qiagen). Using a NanoDrop with a 260/280 nm ratio, we measured the quality and purity of the extract. Using RT–PCR and primer sets using an iScript™ cDNA synthesis kit (Applied Biosystems, Carlsbad, CA) according to the manufacturer’s instructions, cDNA was reverse-transcribed from 0.1 to 0.5 µg of total RNA. Finally, real-time PCR (RT–PCR) was conducted using SYBR Green and an Applied Biosystems 7500 Fast RT–PCR system (Carlsbad, CA) with the gene-specific primers shown in Table 1 and was carried out over 40 cycles at 95 °C for 20 s, 58 °C for 30 s, 95 °C for 15 s, and 60 °C for 30 s. We calculated the relative amount for each gene transcript using the 2-ΔΔCT method and normalized by referencing to the gene GAPDH.

### 4.6. Statistical Analysis

The data were analyzed using GraphPad Prism version 9. A one-way analysis of variance, followed by Tukey’s multiple comparison, was used for the statistical comparisons. All the values are presented as the mean ± standard deviation (SD). Significance was set at a *p* value < 0.05.

## 5. Conclusions

To our knowledge, this is the first study to investigate the intergenerational effects of FB1 on ovarian structure and function. We demonstrate that exposure to high doses of FB1 increases the ovary weight index, in a manner similar to that observed in polycystic ovary syndrome. Additionally, early exposure to high doses of FB1 reduces the number of primordial follicles, which may lead to early ovarian aging among female subjects of both the first and second generations. Moreover, it reduces the fertility rate of the first generation by altering secondary and tertiary follicle growth and development, leading to their degeneration. This alteration may be due to the downregulation of the CYP19 and ESR2 genes. However, the female subjects of the second generation have a limited number of growing follicles compared to the controls, in accordance with the normal expression of the CYP19 gene, and increased mRNA level of ESR2. Specifically, the hallmark histological feature of the ovary from the high-dose treatment group of F2 female rats was the appearance of vacuolar structures within the oocyte and between the granulosa cell layers, concomitant with the significant increase in the autophagic marker LC3. These findings suggest that autophagy may contribute to fertility recovery among female subjects of the second generation due to the histopathological and molecular alterations caused by the early-life effects of FB1. Thus, understanding the interaction of autophagy with damaged cells under the effect of toxicants may become increasingly important in improving infertility therapy among infertile female subjects.

## Figures and Tables

**Table 1:** Primers for the real-time RT–PCR.

Gene Symbol	Sequences
***GAPDH***	F: GCATCTTCTTGTGCAGTGCC R: GATGGTGATGGGTTTCCCGT
***LC3***	F: TGTTAGGCTTGCTCTTTTGG R: GCAGAGGAAATGACCACAGAT
***CYP19A1***	F: GCAACAGGAGCTATAGATGAAC R: AGGCACGATGCTGGTGATG3
***Esr2***	F: GAAGCTGAACCACCCAATGT R: CAGTCCCACCATTAGCACCT
***Gdf9***	F: GATGTGACCTCCCTCCTTCA R: GCCTGGGTACTCGTGTCATT
***Igf1***	F: CCGCTGAAGCCTACAAAGTC R: GGGAGGCTCCTCCTACATTC

## Data Availability

The data that support the findings of this study are available from the corresponding author (Abdel Halim Harrath) upon reasonable request.
